# All-fiberized 1840-nm femtosecond thulium fiber laser for label-free nonlinear microscopy

**DOI:** 10.1364/BOE.495879

**Published:** 2023-08-09

**Authors:** Duanyang Xu, Konstantinos N. Bourdakos, Anna Crisford, Peter Johnson, Ibrahim Abughazaleh, Panuwat Srisamran, Richard O. C. Oreffo, Sumeet Mahajan, David J. Richardson, Lin Xu

**Affiliations:** 1 Optoelectronics Research Centre, University of Southampton, Southampton, SO17 1BJ, UK; 2 Institute for Life Sciences, University of Southampton, Southampton, SO17 1BJ, UK; 3 School of Chemistry, University of Southampton, Southampton, SO17 1BJ, UK; 4 Human Development Health, Faculty of Medicine, Southampton, SO16 6YD, UK

## Abstract

We report an all-fiberized 1840-nm thulium-fiber-laser source, comprising a dissipative-soliton mode-locked seed laser and a chirped-pulse-amplification system for label-free biological imaging through nonlinear microscopy. The mode-locked thulium fiber laser generated dissipative-soliton pulses with a pre-chirped duration of 7 ps and pulse energy of 1 nJ. A chirped-pulse fiber-amplification system employing an in-house-fabricated, short-length, single-mode, high-absorption, thulium fiber delivered pulses with energies up to 105 nJ. The pulses were capable of being compressed to 416 fs by passing through a grating pair. Imaging of mouse tissue and human bone samples was demonstrated using this source via third-harmonic generation microscopy.

## 1. Introduction

Nonlinear optical microscopy using short-pulsed fiber lasers has been widely used for biological imaging research because it allows the minimal invasion, fast speed and high resolution based on various well-established techniques [1,2]. Femtosecond lasers from ytterbium and neodymium fiber systems operating in the near infrared (NIR) region at wavelengths around 1 µm have shown good performances in biological imaging based on two-photon excitation microscopy (TPM) and second-harmonic generation (SHG) microscopy [3,4]. Compared to TPM and SHG microscopy, higher-order nonlinear optical microscopy, such as three-photon excitation microscopy (3PM) and third-harmonic generation (THG) microscopy, provide high signal-to-background ratios and deep penetration [5,6] when using femtosecond excitation pulses in the short-wave infrared (SWIR) region from 1700nm to 1860nm. This is due to this wavelength region providing the window in which the sum of absorption and scattering is minimized from most biological tissues. Solid-state laser sources based on optical parametric oscillator or amplifier techniques involving high-power NIR femtosecond Ti:sapphire lasers and nonlinear frequency converters have been used to demonstrate with good achievements in such higher-order nonlinear optical microscopies [7,8]. Compared to such complex and bulky solid-state lasers, fiber lasers offer great advantages of high efficiency, good compactness and robustness, and good beam quality. Frequency conversion of 1.5-µm femtosecond erbium fiber lasers by soliton self-frequency shift inside a nonlinear optical fiber can generate wavelengths within this SWIR region. However, this technique has a relatively low energy-conversion efficiency, and the output wavelength and pulse energy are highly sensitive to the coupling efficiency into the nonlinear fiber [9,10].

Thulium-doped fiber (TDF) exhibits a broad emission spectrum, spanning from 1600 nm to 2100 nm covering the entire SWIR region and hence represents an attractive gain medium for femtosecond fiber lasers that have potential in such imaging applications [11,12]. Generally, silica-glass based TDF lasers operate at wavelengths beyond 1900nm, where the emission cross sections of Tm^3+^ ions in silica are large and high optical gain can be obtained. A TDF laser system generating 297 fs pulses at 1925nm with pulse energy up to 1.27 µJ has been demonstrated [13]. However, it is challenging to operate the short-pulsed TDF laser at the SWIR wavelengths with a high pulse energy due to the quasi-three-level nature of the thulium laser and the need for careful management of nonlinearity, dispersion and spectral filtering in such laser system. Furthermore, short-pulse amplification at such a wavelength is compromised by undesired spectral and temporal distortions due to the combination of nonlinearity and anomalous dispersion in typical silica-glass based TDFs. The powers at which these distortions start to be limiting can be increased by careful design of the silica-glass TDF amplifiers to provide high gain over a short device length. This approach allows the generation of high-quality, high-energy pulses with sufficient peak powers for nonlinear microscopy applications from a fully fiberized laser system. A mode-locked (ML) TDF laser operating at 1746nm has been reported but with a low power efficiency generating pulse energy of only 0.2 nJ, based on nonlinear polarization rotation (NPR), and which usually results in poor self-starting of the ML laser [14]. By using a dispersion engineered TDF, a ML laser operating in the 1.7-1.8-µm region has been demonstrated and a pulse energy of 128 nJ was obtained in a fiber amplifier system [15]. However, it required a complicated fiber geometry design and fabrication process that could prevent wider application. Instead of using silica-glass TDFs, a 1820-nm fiber laser with pulse energy of 1.1 µJ was developed by using fluoride-glass TDFs and 3PM imaging of mouse brain by using such laser was demonstrated [16]. However, it is difficult to splice the fluoride-glass TDFs used in that system to other silica-glass fiber components, hence the system is bulky and involves significant free-space alignment. In addition, special preparation of biological samples is required, in which fluorescent protein labels are transfected to facilitate the 3PM imaging. Third-harmonic generation microscopy is label-free and provide information on biological structures in tissue. Here we report the development of an all-fiberized 1840-nm femtosecond laser system based on standard silica-glass TDFs and demonstrate a proof-of-principle study of its application in label-free THG microscopy of biological samples.

## 2. Fiber laser source

### 2.1 Mode-locked thulium-doped fiber laser

**Fig. 1.**
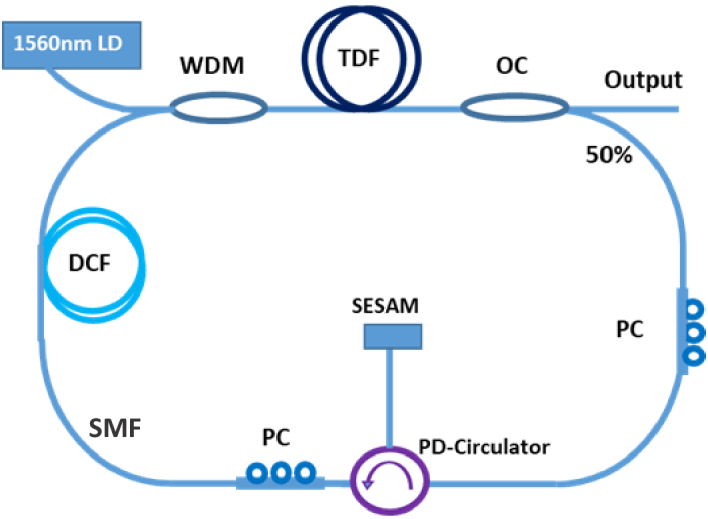
Schematic of the mode-locked thulium fiber laser cavity. WDM: wavelength-division multiplexer; TDF: thulium doped fiber; OC: output coupler; SMF: single mode fiber; PC: polarization controller; PD-Circulator: polarization dependant circulator; DCF: dispersion compensate fiber; LD: laser diode; SESAM: semiconductor saturable absorber mirror.

A schematic of the ML cavity is shown in Fig. 1. A unidirectional ring cavity consisting of a length of single-mode TDF (OFS, TmDF200) as the gain medium, pumped by a 1560-nm diode laser (Princeton Lightwave, DEI14919) through a 1560-nm/1840-nm wavelength-division multiplexer (WDM). The combination of the high-gain TDF with the laser diode pump source enables a ML in a compact format. A semiconductor saturable absorber mirror (SESAM) with a non-saturable reflection of 60% and a modulation depth of 20% (Batop, SAM-1920-36-10ps) was attached to the fiber end of a polarization-dependent (PD) circulator, which ensured unidirectional oscillation in the cavity. To compensate for the anomalous dispersion of the single-mode fibers (SMF) in the cavity, a length of dispersion-compensating fiber (DCF) (Coherent, UHNA4) was employed to provide an overall net normal dispersion for the targeted dissipative-soliton mode-locking operation. Two in-line polarization controllers (PC) were used to adjust the polarization state of the intra-cavity pulses generating Lyot filtering effects and stabilizing the mode locking in the cavity [13]. An output coupler with a 50% transmission was used to extract the laser output.

**Fig. 2.**
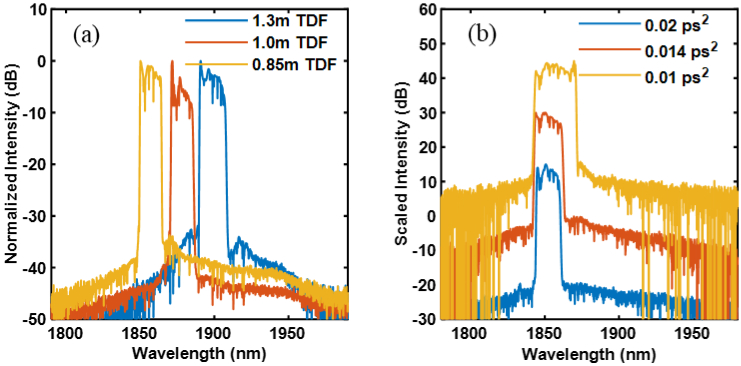
Output spectrum of the mode-locked fiber laser with (a) varying the length of TDF and (b) changing the net cavity dispersion.

The TDF had an absorption coefficient of ∼20 dB/m at the pump wavelength, and a length of 1.3 m was initially chosen in order to favor laser operation at the short wavelength end of the thulium emission bandwidth. The total lengths of the SMF and DCF used in the cavity were measured to be 6.2 m and 5 m, respectively. Considering the group-velocity dispersion of the fibers used in the cavity at the laser wavelength (SMF: −0.06 ps^2^/m, TDF: −0.02 ps^2^/m, DCF: + 0.093 ps^2^/m), the net cavity dispersion was estimated to be 0.07ps^2^. With proper adjustment of the PCs in the cavity, self-starting, single-pulse, stable mode locking was observed at a pump power of 150 mW. The output pulses had a repetition rate of 16.36 MHz, which was consistent with the cavity length. The output spectrum was measured showing a central wavelength at 1900nm. In order to characterize the influence on the operating wavelength and optimize the gain fiber length, the mode locking was tested by shortening the TDF length and the corresponding output spectra are shown in Fig. 2(a). The central wavelength of the ML laser could be effectively shortened by using a shorter length of TDF due to the quasi-three level system of the thulium ions [17]. The influence of the net dispersion on the output spectral bandwidth was also characterized by fixing the TDF length while changing the length of DCF. Figure 2(b) shows the results, in which a smaller net normal dispersion resulted in a broader output spectrum that would generally produce shorter pulses after de-chirping.

**Fig. 3.**
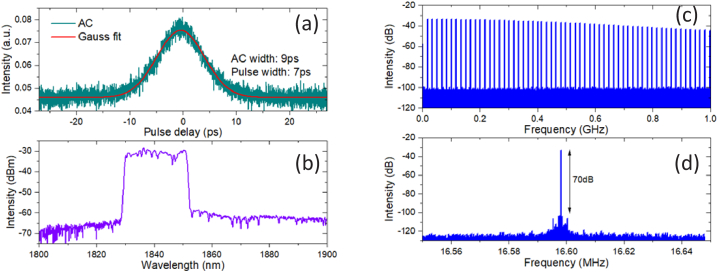
Autocorrelation trace (AC) and spectrum measurement (a), (b) and RF spectrum measurement (c), (d) of the 1840-nm mode-locked fiber laser.

In order to achieve the target operating wavelength of 1840nm, a TDF length of 0.7 m and net cavity dispersion of ∼0.01 ps^2^ were chosen (SMF length: 6.9 m, DCF length: 4.7 m) for the ML laser. The mode locking output spectrum had a central wavelength of 1840nm and 10-dB spectral bandwidth of 22 nm. The output pulse was measured to have an autocorrelation width of 9 ps corresponding to a Gaussian pulse with a width of 7 ps, as shown in Fig. 3(a). 17 mW of average output power was obtained at the maximum available pump power of 200 mW from the 1560-nm LD. The RF spectrum measurement of the output pulses shows a fundamental frequency of 16.6 MHz and an optical signal-to-noise ratio of 70 dB, as shown in Fig. 3(b).

### 2.2 Thulium-doped fiber amplifiers

After an isolator the output pulses from the mode-locked laser were attenuated to an average power of 5 mW and passed through a 23-m-length DCF (Coherent, UHNA4) to stretch the pulse width to 18 ps and avoid nonlinear distortion in the following fiber amplifiers. The first fiber amplifier consisted of a 1-m-length of TDF (OFS, TmDF200) pumped by a home-built 1565-nm Erbium-doped fiber laser (EDFL). At a maximum pump power of 300 mW, the first fiber amplifier generated an average power of 50 mW at the output.

**Fig. 4.**
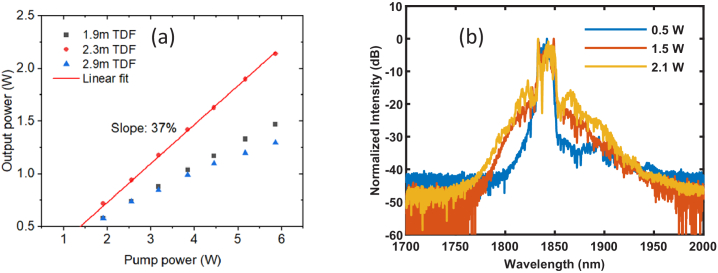
Output power measurement (a) and spectrum measurement (b) of the second fiber amplifier with the 9-µm core diameter TDF.

In the second fiber amplifier, single-mode TDFs with bigger cores were required to realize Watt-level output powers without nonlinear distortion. Firstly, a commercial TDF with a core diameter of 9 µm (Coherent, TSF-9/125) and an absorption of ∼12 dB/m at 1550 nm was used and pumped by a 1550-nm EDFL with a maximum available power of 10 W. To optimize the gain fiber length for the second fiber amplifier, three different lengths of the TDF (1.9 m, 2.3 m and 2.9 m) were selected and tested for their output power performance. As shown in Fig. 4, the 2.3-m-TDF amplifier provided a power slope efficiency of 37% and a maximum output power of 2.1 W at a pump power of 6 W. However, the output spectrum showed significant nonlinear distortions with increasing power, as shown in Fig. 4, which is probably due to modulation instability in the anomalous dispersion region.

**Fig. 5.**
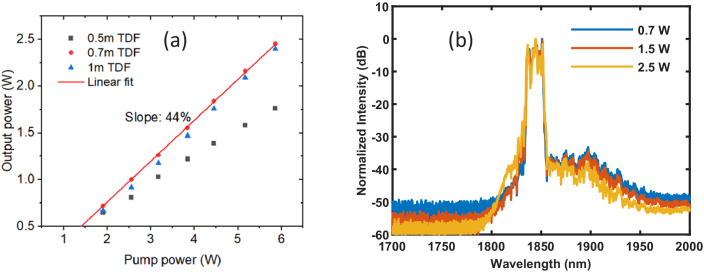
Output power measurement (a) and spectrum measurement (b) of the second fiber amplifier with the 10-µm core diameter TDF.

In order to eliminate the nonlinear distortion, an in-house designed and fabricated single-mode TDF, which had a core size of 10 µm and a higher absorption of ∼37 dB/m at the pump wavelength, was employed to allow a shorter length of TDF in the second fiber amplifier [18]. By characterizing the amplifier power performance with different lengths of TDF (0.5 m, 0.7 m and 1 m), as shown in Fig. 5, an optimum length of 0.7 m was chosen for the fiber amplifier. The output power increased with the pump power at a slope efficiency of 44% and reached a maximum of 2.5 W at a pump power of 6 W, corresponding to a maximum pulse energy of 150 nJ. In comparison to the previous results, the output spectrum did not exhibit obvious nonlinear distortion at such power levels.

**Fig. 6.**
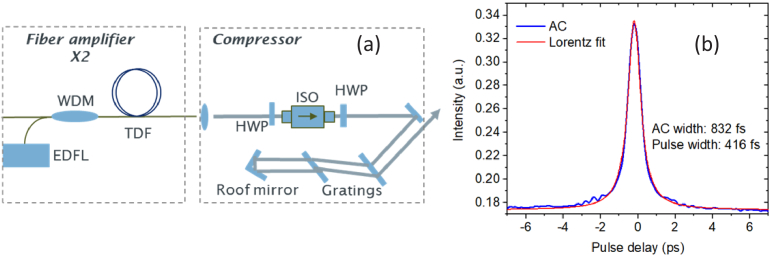
Schematic of the fiber amplifier and pulse compressor (a) and autocorrelation measurement of the compressed pulse (b).

The collimated output beam passed through a free-space isolator and was then delivered into a pulse compressor, which consisted of a pair of fused silica transmission gratings with a groove density of 560 lines/mm and a roof mirror, as shown in Fig. 6(a). Due to the non-polarization maintaining fibers used in the amplifiers, the combined loss of the polarization-sensitive isolator and pulse compressor was measured to be around 3 dB. The autocorrelation measurement of the compressed pulses is shown in Fig. 6(b), which exhibited a pulse width of 416 fs by a Lorentzian fit. Further compression to a shorter pulse was not possible, likely due to a combination of (uncompensated) higher-order dispersion, and nonlinear effects within the fibers and amplifiers comprising the system.

## 3. Third-harmonic generation microscopy

**Fig. 7.**
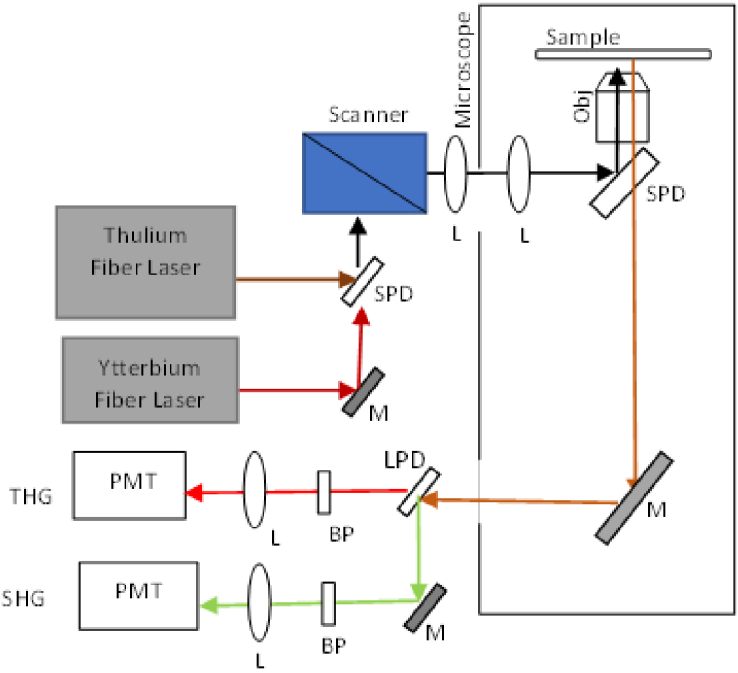
Schematic of the imaging system. PMT: photo-multiplier tube, L: lens, BP: band pass filter, M: mirror, LPD: long pass dichroic, SPD: short pass dichroic, Obj: objective lens, THG: third-harmonic generation, SHG: second-harmonic generation.

The imaging was performed with a custom-built inverted microscope based on a Nikon Ti Eclipse frame, which was equipped with a pair of galvanometer mirrors for laser scanning. As illustrated in Fig. 7, the output beam of the thulium fiber laser was coupled into the scanner, and after passing through a pair of lenses (scan lens and tube lens) it was reflected by a short-pass dichroic (excitation dichroic) beam splitter and focused on the samples through an infinity corrected objective. The total transmission of the optics within the microscope for the laser beam was approximately 15%. The majority of the loss was in the microscope objective which showed <40% transmission at 1840nm. The THG signal was collected in the back-scattering (epi-detection) geometry from the same objective and delivered through optics comprising of the excitation dichroic, mirrors, lenses, and short-pass filters, as well as a narrow band-pass filter to a photomultiplier tube (PMT) detector. The scanner and the detector were interfaced with a DAQ-PCI6110 to a desktop computer. The laser scanning and the acquisition were controlled with Scanimage 6.1 (Vidrio Technologies) [19]. All the image acquisitions were performed with a 20**×** objective lens with a numerical aperture (NA) of 0.75. The pixel resolution of each image was 512 **× **512 which, for the objective and the scanning settings that we have used, corresponded to a field of view of 250 µm **×** 250 µm, while the pixel dwell time was 16 µs.

For one of the samples shown in Fig. 11, SHG images were concurrently acquired with the THG images to demonstrate complementary information about the tissues. Multimodal multiphoton microscopy to acquire both SHG and THG by using the thulium fiber laser was not undertaken, due to the lack of suitable PMTs having good response at the SHG wavelength of the thulium fiber laser, but in principle could work. In this case, an ytterbium fiber laser (1031 nm, 2 ps, 80 MHz), whose output had been made colinear with the thulium fibre laser output, was used for the excitation of the SHG. The THG and SHG signals were spectrally separated with a long pass dichroic beam-splitter in the detection branch, with the SHG signal being reflected and passed through another band-pass filter centered at 520 nm, with a full width at half maximum (FWHM) of 40 nm, towards a second PMT.

**Fig. 8.**
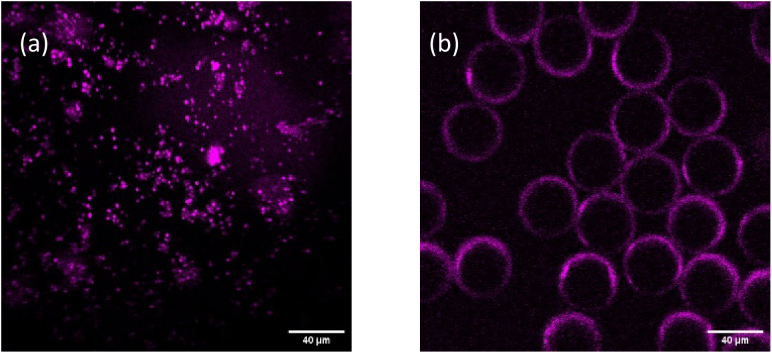
THG imaging of (a) BaTiO3 nanocrystals and (b) polystyrene beads. The scale bars are 40 µm

Firstly, the THG imaging capabilities of the apparatus were tested by imaging agglomerates of BaTiO_3_ nanocrystals (Fig. 8(a)), which are known for their high third order susceptibility [20]. Nanocrystals with diameters between 50 to 100 nm, in a powder form, were dispersed on a microscope slide and covered with a glass coverslip (0.17 mm thickness). As described above, a band-pass filter with a central wavelength of 620 nm and a FWHM of 20 nm was placed in front of the detector. The THG signals were verified by using different filters to exclude the spectrum ranging between 610 to 629 nm; no signal was observed, which strongly suggested that the detected signals when using the 620 nm band-pass filter were indeed from the THG of the excitation. The same tests were applied during the image acquisition for all the other samples presented in this work. In addition, a sample consisting of 40-µm-diameter polystyrene beads was chosen to test the THG imaging system functionality. Clear imaging showing strong signals at the boundaries of the polystyrene spheres and negligible signal from the bulk was achieved, as presented in Fig. 8(b), which is known due to the sensitivity of THG to interfacial surfaces [21,22].

**Fig. 9.**
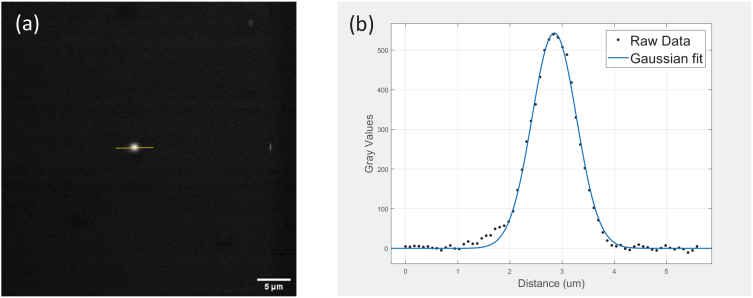
(a) A representative image of an isolated THG active spot on a sample made with sparsely dispersed 50-100 nm BaTiO3 nanocrystals. The scale bar is 5 µm. (b) The intensity profile (black dots) across a line (as shown in Figure a) and a Gaussian fit (blue). The full width at half maximum of the Gaussian fitted peak is found to be 0.87 µm.

An evaluation of the lateral point spread function (PSF) of the THG imaging system is shown in Fig. 9. It was acquired by imaging a sample containing sparsely dispersed 50-100 nm BaTiO_3_ nanocrystals on a microscope slide and covered with a standard coverslip. The sizes of the nanocrystals were as per supplier specifications and used after vigorous shaking to break any agglomerates and used as such only to estimate the PSF. Figure 9(a) shows a typical image acquired on this sample, which is very close to the average size of nanoparticle images observed. Figure 9(b) shows the profile plot along the yellow line (minus a background offset) in Fig. 9(a) (black dots) and the corresponding Gaussian fit (blue curve) which yielded a FWHM of 0.87 µm. The latter implies that the width of the real PSF is around or lower than 0.87 µm.

In order to demonstrate the biomedical imaging application of the Thulium fiber laser, a number of fixed soft and hard tissue samples were prepared and tested. These images were acquired by using the optical lay-out and imaging settings described above. Regarding the preparation of the mouse samples, all procedures were carried out in accordance with the Animals (Scientific Procedures) Act 1986 set out by the UK Home Office. The human samples were purchased from a commercial supplier (Amsbio, USA).

**Fig. 10.**
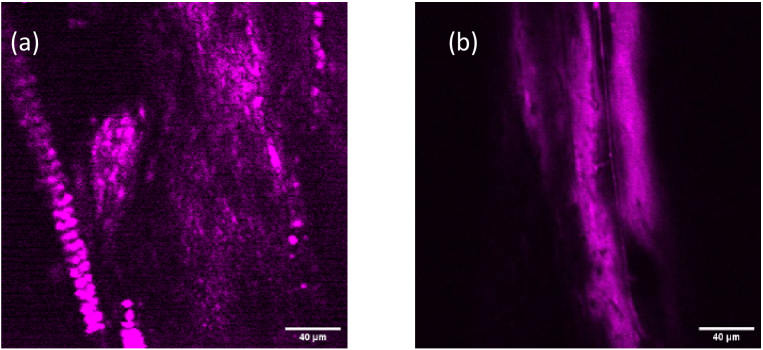
THG imaging of (a) section of rat tail tendon and (b) section of rat tail with skin removed. The scale bars are 40 µm

In Fig. 10(a) an image of a rat tail tendon from the THG microscopy is shown where the array of dotted and fibrillar areas are likely to be collagen bundles at the interface with the proteoglycan matrix, which has strong THG signals. A part of a rat-tail from which the skin has been removed and includes muscle tissue and blood vessels is shown in Fig. 10(b). Vascular canals and the surrounded collagen matrix can be clearly observed from this image.

**Fig. 11.**
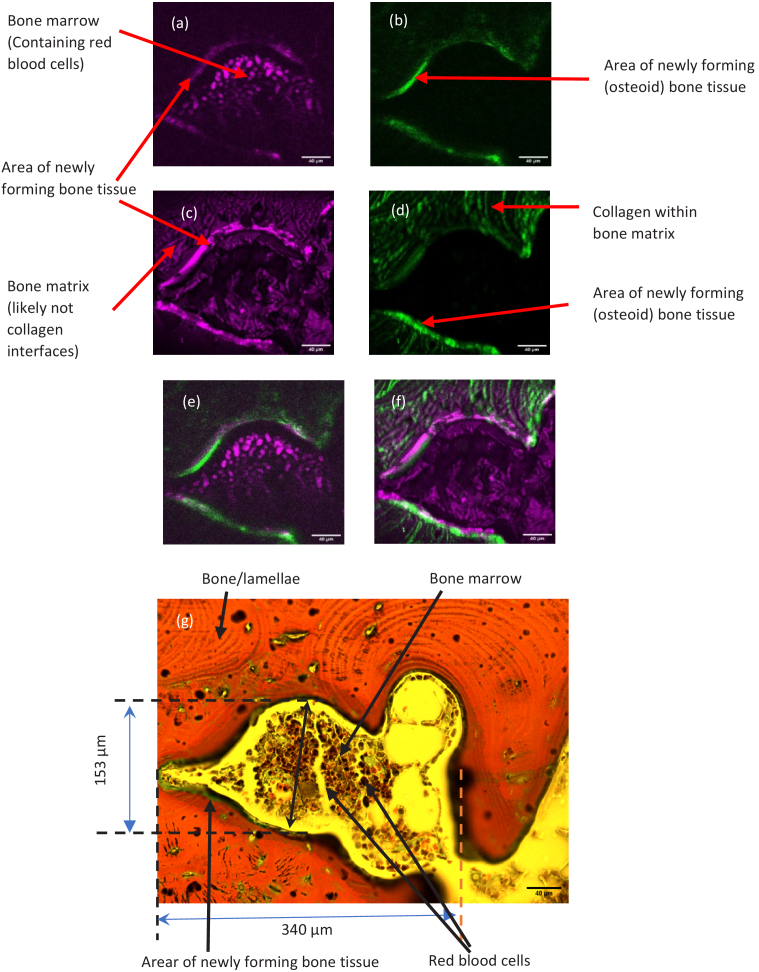
THG (a) and SHG (b) images of the superficial human bone layer, THG (c) and SHG (d) of the deep bone layer (21 µm depth). Composite images THG (magenta) and SHG (green) image of surface (e) and deep (f) layers of (a), (b) and (c), (d) respectively. Bright-field microscopy image of the stained human bone sample (g). The scale bars are 40 µm

THG and SHG images of a section of human rib-cage bone sample, which were acquired concurrently by using the two independent lasers, are shown in Fig. 11. The respective THG and SHG images of the bone sample at the surface layer are depicted in Fig. 11(a) and Fig. 11(b). The same section of the sample was stained with Goldner’s trichrome stain to aid in identifying the bone structures in the THG and SHG images Fig. 11(g). The part of the imaged section is defined with the dotted line. Red areas with concentric circles indicate decalcified bone and lamellae within the image. The yellow structure in the centre of the sample is the bone marrow including red blood cells which are stained as red. A darker line around the bone marrow circumference, staining in green, is a newly forming bone tissue containing newly formed osteoid/collagen. The staining observed correlated with the published data elsewhere [23]. As expected, the THG image displayed a granular structure in the bone marrow compartment, likely red blood cells. Interestingly, part of the surrounding inorganic matrix deposit presented with high contrast, which is likely to be the bone matrix interface. SHG images of collagen fibers in the bone matrix were observed with excellent contrast and an absence of signal in the bone marrow compartment. In Fig. 11(c), THG imaging of bone layer at a depth of 21 µm in the same section highlights potentially the non-collagen interfaces in both bone and bone marrow compartment. Large solid dots in Fig. 11(a) are also observed, which are believed to be erythrocytes (red blood cells). On the periphery of the bone marrow compartment, the bone mineral matrix can be clearly delineated using THG imaging and corresponds to the newly mineralizing bone tissue (stained green in Fig. 11(g)). The corresponding SHG image in Fig. 11(d) demonstrates, the surrounding collagen matrix again highlighting the complementarity of THG and SHG imaging. The composite image of the bone sample at the surface layer is shown in Fig. 11(e), where the THG is depicted in magenta and SHG is depicted in green. Similarly, the composite image of Fig. 11(c) and Fig. 11(d) is shown in Fig. 11(f) using the same colours for the THG and SHG.

Preliminary studies of multiphoton microscopy based on THG by using the all-fiberized thulium fiber laser have thus shown good potential in biological imaging application. Further systematic characterization of the multiphoton microscopic imaging and exploration of deep penetration by using the all-fiberized 1840-nm thulium fiber laser with different objective lenses and filters is underway.

## 4. Summary

In summary, an all-fiberized 1840-nm thulium fiber laser source comprising of a dissipative-soliton mode-locked seed laser and a chirped-pulse-amplification system has been developed and been applied for label-free biological imaging through nonlinear microscopy. The mode-locked thulium fiber laser generated pre-chirped pulses with duration of 7 ps and pulse energy of 1 nJ. A chirped-pulse fiber amplification system was built, where commercially available low-absorption and in-house fabricated high-absorption single-mode thulium fibers were employed and compared, which delivered pulses with energies up to 105 nJ. The pulses were compressed to 416 fs by passing through a grating pair. The THG microscopy imaging capability of the laser system was demonstrated on BaTiO_3_ nanocrystals and biological samples including mouse and human bone tissue and THG provided complementary information to other imaging techniques such as SHG

## Data Availability

Data are available at [24].
